# Highly Stable Liquid Metal-Based Pressure Sensor Integrated with a Microfluidic Channel

**DOI:** 10.3390/s150511823

**Published:** 2015-05-21

**Authors:** Taekeon Jung, Sung Yang

**Affiliations:** 1 Department of Medical System Engineering, GIST, Gwangju 500-712, Korea; E-Mail: taekeonjung@gist.ac.kr; 2 School of Mechatronics, GIST, Gwangju 500-712, Korea

**Keywords:** pressure sensor, liquid metal, galinstan, microfluidic, viscosity

## Abstract

Pressure measurement is considered one of the key parameters in microfluidic systems. It has been widely used in various fields, such as in biology and biomedical fields. The electrical measurement method is the most widely investigated; however, it is unsuitable for microfluidic systems because of a complicated fabrication process and difficult integration. Moreover, it is generally damaged by large deflection. This paper proposes a thin-film-based pressure sensor that is free from these limitations, using a liquid metal called galinstan. The proposed pressure sensor is easily integrated into a microfluidic system using soft lithography because galinstan exists in a liquid phase at room temperature. We investigated the characteristics of the proposed pressure sensor by calibrating for a pressure range from 0 to 230 kPa (*R*^2^ > 0.98) using deionized water. Furthermore, the viscosity of various fluid samples was measured for a shear-rate range of 30–1000 s^−1^. The results of Newtonian and non-Newtonian fluids were evaluated using a commercial viscometer and normalized difference was found to be less than 5.1% and 7.0%, respectively. The galinstan-based pressure sensor can be used in various microfluidic systems for long-term monitoring with high linearity, repeatability, and long-term stability.

## 1. Introduction

Pressure is one of the key factors in microfluidic systems, which has been intensively studied in various applications. Precise pressure control can provide large benefits in achieving successful results because the fluid flow in microfluidic systems, in many cases, is driven by pressure [1,2,3,4,5,6,7,8]. In particular, a precisely controlled flow rate is highly desired in biological applications based on microfluidic systems. For instance, biological samples are focused, separated, and transferred to a desired location in pressure-driven microfluidic systems [1,2,3]. Moreover, a proper cellular microenvironment for cell or bacteria culture [4,5], which usually requires long-term experiments, can be well maintained by providing media and real-time monitoring of pressure. In the study of cell behavior [6,7,8], accurate pressure is essential for applying mechanical force and monitoring the occurrence of unexpected events. In addition to biological applications, pressure plays an important role in medical systems, such as in intraocular pressure (IOP) sensors for glaucoma [9,10], in bladder pressure sensors [11], and in cardiovascular pressure sensors [12,13]. These sensors are integrated into an intraocular lens, stents, or catheters, and perform 24 h monitoring of various clinical parameters. Therefore, high-performance pressure sensors are required to achieve desired results in various applications; thus, diverse pressure-sensor types have been widely reported, such as in optical or electrical measurement methods.

Optical-sensor types that use membrane- or beam-deflection intensity [14,15,16], interferometry [17,18,19], and fluid interface [20,21] are famous for their high sensitivity, stability, and easy integration in microfluidic systems. However, the optical measurement type is impractical because of the requirement for large-scale instruments to obtain images and the complicated analysis steps to estimate the pressure. In the case of the interface-monitoring-type, the experimental results are affected by the reference fluid, which directly contacts the sample fluid. Electrical measurement types, such as capacitive [22,23,24] and resistive [25,26,27,28] pressure sensors, have been intensively investigated owing to their simple measurement methods compared with the optical type. Capacitive pressure sensors estimate pressure by monitoring the signal variation according to the gap change between the two electrodes located at the top and bottom layers. Meanwhile, resistive sensors measure the electrode-resistance change caused by membrane deflection. These sensor types are promising as tactile sensors because of their high sensitivity and high spatial resolution with the use of an array-type sensor. However, a complicated fabrication process, such as metal deposition and etching, is required; thus, this sensor type is unsuitable for integration in microfluidic systems. Furthermore, the sensor can possibly be damaged due to a large deflection or unexpected impact as this sensor type generally uses mechanically brittle materials, e.g., silicon, carbon fiber, and conductive polymer.

In the current paper, we report a liquid metal-based pressure sensor, embedded in a microfluidic system. We adapted a resistive-type pressure sensor owing to its simple measurement method and simple instrumentation. To improve the few limitations of resistive-type pressure sensors, we utilized a liquid metal called galinstan. Recently, galinstan, which is a eutectic alloy of gallium, indium, and tin, has been widely employed in various applications involving micro electrodes [29,30,31], mixers [32], and pumps [33] because it exists in the liquid phase at room temperature (melting point is −19 °C), has high conductivity, and is non-toxic. We investigated the performance of the proposed pressure sensor in terms of linearity, reliability, and repeatability. We also proposed its application as a microviscometer by measuring the viscosity of various Newtonian and non-Newtonian fluid samples. Liquid metal-based pressure sensors can provide high stability for long-term experiments, simple measurement and fabrication methods, and can easily be integrated in various lab-on-a-chip systems. Moreover, the proposed pressure sensor is highly reversible without showing damage, even at high pressure.

## 2. Experimental Section

### 2.1. Device Design

**Figure 1:**
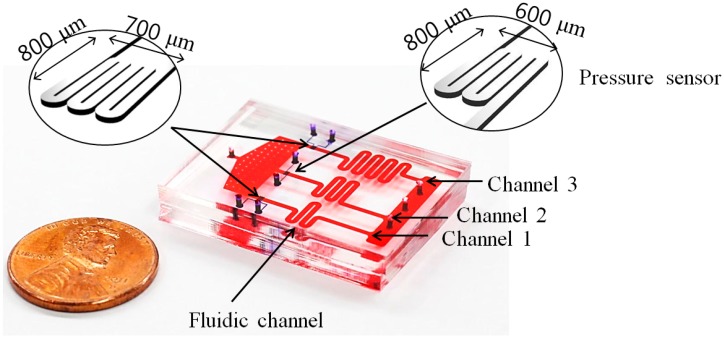
Schematic of the device. The pressure sensor integrated in the microfluidic device consists of three PDMS layers: sensor, thin-film, and fluidic-channel layers. The sensor channel is filled with galinstan.

Figure 1 shows the proposed pressure sensor, integrated into a microfluidic system. The device consists of polydimethylsiloxane (PDMS) layers: fluidic channel (bottom layer), PDMS thin membrane (middle layer), and pressure-sensor channel (top layer). The height and width of the fluidic channel are 20 and 800 μm, respectively. The device has three channels for simultaneous measurement of the sample fluid viscosity under three different shear-rate conditions. Since the shear rate depends on the flow rate, the three different channels were designed to have different lengths using a serpentine pattern for different shear-rate conditions from the same inlet flow rate. All outlets of each channel were connected to make an equal pressure. When the channel width and height were fixed, the flow rate increased with the decrease in the channel length due to the decrease in hydraulic resistance. The galinstan-filled pressure sensor was located at the top layer. The width and height of the sensor channel were 20 and 100 μm. A 15-μm-thick PDMS membrane was placed between the top and bottom layers. When the membrane is subjected to pressure, it is deflected in the upward direction, proportional to the flow rate. Thus, the electrical resistance of galinstan, which fills the pressure sensor, changes because of the decrease in the channel area; and, hence, the pressure can be estimated by measuring the resistance change.

The top and bottom layers of the proposed device were fabricated using a standard soft lithography process. A negative photoresist (SU-8 2025) was spin-coated on a bare silicon wafer for 40 s at a speed of 4000 rpm. After soft baking, the SU-8-coated silicon wafer was exposed to an ultraviolet light for 10 s using a mask aligner and, consequently, developed in an SU-8 developer. A PDMS (Sylgard 184, Dow Corning) and curing agent mixture, at a 10:1 weight ratio, was poured onto the SU-8 patterned wafer. After degassing in a vacuum chamber for 20 m, a PDMS replica mold was realized by curing in an oven at 80 °C for 90 m. For the PDMS thin membrane, the degassed PDMS mixture was spin-coated on a slide glass at a speed of 2500 rpm, and the top layer was then bonded on the PDMS thin membrane using oxygen plasma surface treatment. After peeling off the thin membrane, the bottom layer was bonded to it. The entire device was realized by filling the galinstan into a pressure-sensor channel and a wire was connected to the terminals of the sensor channel to measure the resistance change.

### 2.2. Pressure Measurement

**Figure 2:**
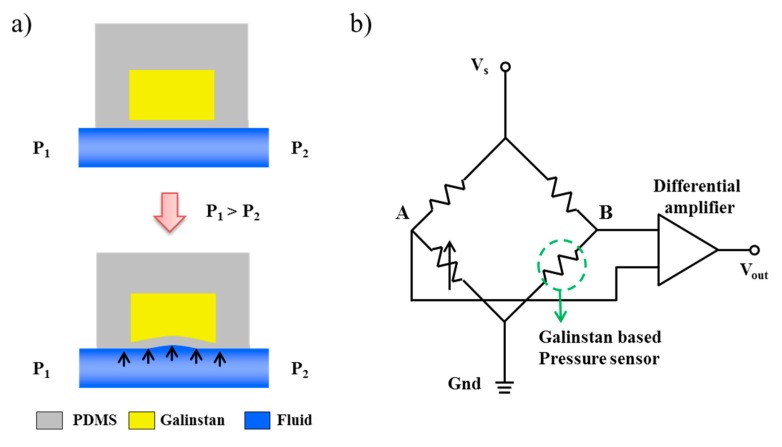
Working principle of a galinstan-based pressure sensor. (**a**) When the membrane is subjected to pressure, the electrical resistance of the pressure sensor gets increased due to reduction in the cross-sectional area; (**b**) Schematic of the Wheatstone bridge circuit. The pressure is estimated by measuring the voltage difference between nodes A and B.

Figure 2a shows a cross-sectional view of the proposed pressure sensor. Galinstan is introduced in the pressure-sensor channel, while the fluid flows through a fluidic channel located under the membrane. In this study, we utilized the electrical-resistance (*R*_e_) change of galinstan in the pressure sensor to measure the applied pressure. Electrical resistance is proportional to the resistivity and length of the pressure-sensor channel and inversely proportional to the cross-sectional area of the channel according to the electric-resistance theory form given by:

(1) R e=ρ(l/A)

where ρ is the resistivity, l is the length of the pressure sensor, and *A* is the cross-sectional area. The resistivity is constant as a physical property of galinstan, and the channel length is fixed for the employed device. Consequently, the electrical-resistance change is affected by the cross-sectional area change, which is also affected by pressure. At rest, the inlet (*P*_1_) and outlet (*P*_2_) pressures are identical, and the pressure sensor has an initial resistance because the membrane is not deflected. Meanwhile, the resistance of the pressure sensor increases because of the reduction in the channel area when *P*_1_ is higher than *P*_2_ under a certain flow rate. We can estimate the pressure at a specific position in the microfluidic channel by measuring the voltage signal, which is proportional to the resistance of the pressure sensor, using the data acquisition (DAQ) system of the LabVIEW program.

To measure the signal change, we utilized a Wheatstone bridge circuit, which is widely used to measure accurate signal changes using standard resistances. From the Wheatstone bridge, we can estimate the pressure by measuring the voltage change between nodes A and B using a differential amplifier (Figure 2b). The voltage at node B, which is proportional to the resistance of the pressure sensor, increases according to the applied pressure, whereas the voltage at node A always remains constant. Thus, the measured voltage is only affected by the proposed pressure sensor, and the signal change in each pressure sensor is simultaneously measured using a multiplexer.

## 3. Results

### 3.1. Calibration

**Figure 3:**
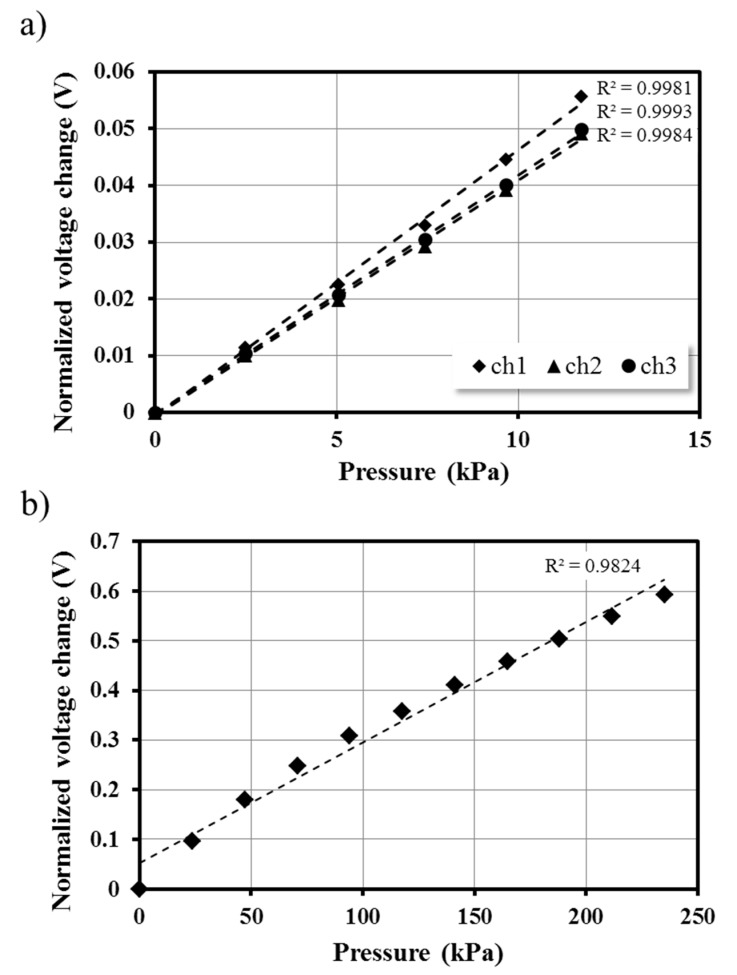
Calibration of the pressure sensors. Calibration results of each pressure sensor in the proposed device with pressure of (**a**) 12 kPa and (**b**) 230 kPa. From the linear regression analysis, the calibration results show large linearity (*R*^2^ > 0.999 for 12 kPa, and *R*^2^ > 0.982 for 230 kPa).

To investigate the characteristics of the pressure sensor, we performed its calibration at different flow rates. Deionized (DI) water was injected through the inlet of the fluidic channel using a syringe pump (neMESYS, Cetoni GmbH, Germany). The inlet flow rate was precisely controlled from 0 to 5 mL/h with a 1-mL/h increment, which can cover the entire shear-rate range for a microviscometer application. Since the voltage change was completely saturated within 1 min at the given flow rate, each flow rate was maintained for 1 min to ensure full deflection of the PDMS thin membrane. The voltage change according to the inlet flow rate was continuously measured using the DAQ system. Figure 3a shows the averaged signal variations in each flow rate. The flow rate was converted to pressure using commercial pressure sensors (24PCDFA6G, Honeywell, Morristown, NJ, USA). Three different Honeywell sensors were connected at exactly the same place in the device where the galinstan-based pressure sensor was located. DI water was infused using a syringe pump at an identical inlet flow rate, which was used in the calibration experiment. The pressure for each flow rate was measured by monitoring the voltage change in the commercial pressure sensors. To evaluate the proposed galinstan-based pressure sensor, a linear regression analysis was implemented. The three different pressure sensors located in each channel showed excellent linearity (*R*^2^ > 0.999) within the pressure range from 0 to 12 kPa. In addition, we carried out calibration using a flow rate of 100 mL/h, which matched a 230-kPa pressure, to investigate the detection limit of the pressure sensor. Figure 3b shows the normalized voltage change for different flow rate. From the linear regression analysis, the result showed high linearity (*R*^2^ > 0.98), and the correlation coefficient (*R*^2^) was more than 0.99 at a pressure range from 0 to 50 kPa. In order to precisely calibrate the pressure sensor, the effect of the change in ambient temperature should be considered. However, the ambient temperature for biological applications is not significantly different from the room temperature; the pressure sensor does not need an additional calibration process, considering the additional deformation may be caused by the change in ambient temperature is negligible for biological applications [34,35].

### 3.2. Long-Term Stability

**Figure 4:**
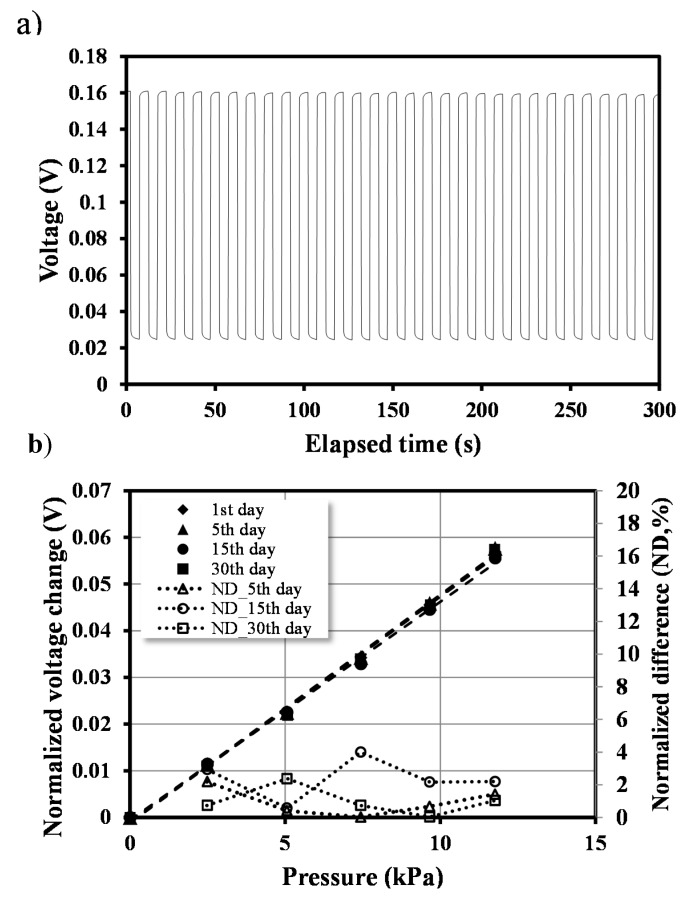
Long-term stability test. (**a**) Pressure of 30 kPa is repeatedly applied to the microfluidic channel using a pneumatic pump at a 5-s interval; (**b**) Calibration is conducted four times for 30 days, and the normalized difference between the initial data and the data measured each day is less than 5%.

In this study, we investigated the long-term stability of the pressure sensor. A highly stable pressure sensor in terms of reliability and repeatability is desired in various microfluidic systems that conduct long-time experiments. For instance, cell- or molecular-based applications generally take several days experimentation to achieve meaningful results. Pressure monitoring is essential to control the applied pressure or to prevent sudden impact, which is undesirable during an entire experiment. A periodic pressure of 30 kPa was applied to the microfluidic channel of the proposed device using a pneumatic pump. The pressure was repeatedly applied and removed at a 5-s interval for 2 h. Figure 4a shows the signal change in Channel 1 according to the applied pressure. When pressure was applied to the proposed device, the response time, which was measured using the time interval between 10% and 90% of the steady state under each pressure, was less than 0.5 s. The average signal change was 158 ± 0.90 mV for 2 h. Furthermore, the calibration was performed four times for 30 days. Figure 4b shows the normalized voltage change for pressure change from 0 to 12 kPa. The normalized measured voltage is defined as the measured voltage under the given pressure minus the measured voltage under the zero pressure. The normalized difference is defined as the following Equation (2):

(2) $$ND_{\_nth}{\;}_{day}=\;|\frac{Normalized\;Measured\;Voltage_{\_nth\;day}-Normalized\;Measeured\;Voltage_{\_1st\;day}}{Normalized\;Measured\;Voltage_{\_1st\;day}}|\times 100$$

The normalized voltage difference was found to be less than 5%. The results show that the proposed device is capable of long-term stable pressure monitoring in various microfluidic applications.

**Figure 5:**
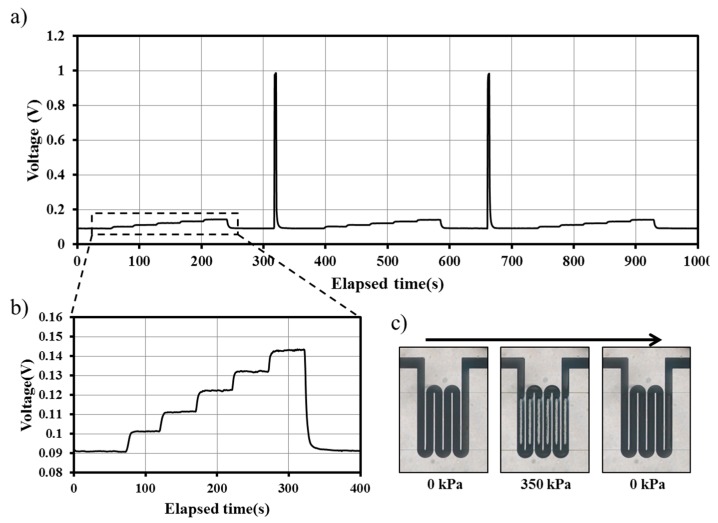
(**a**) Real-time monitoring of signal change. Calibration was repeatedly conducted after applying high pressure (350 kPa); (**b**) Magnified graph of the initial calibration results. The voltage signal increased with the pressure increase from 0 to 12 kPa; (**c**) Time-lapse sequential image obtained by a microscope. Most of the galinstan was expelled to the connection part under 350 kPa of pressure, but the galinstan totally refilled the sensor part immediately after the applied pressure was removed.

To further characterize the pressure sensor, we demonstrate its reversibility by operating it at pressures beyond the detection limit. During the experiment, the voltage change in the pressure sensor at Channel 1 was continuously monitored (Figure 5a). First, we carried out the calibration under a flow rate range from 0 to 5 mL/h (Figure 5b). After calibration, high pressure was applied to the pressure sensor using a 150-mL/h (350 kPa) flow rate for 5 s. The same procedure was repeated 20 times. The calibration results were compared to evaluate the reversibility of the pressure sensor. The difference in each calibration result was less than 5.6%. Figure 5c shows a microscopic image of the pressure sensor, captured in a time-lapse sequence from left to the right side. The images show that galinstan completely refilled the entire sensor channel after the high pressure was removed, although most of the galinstan was expelled to another part of the sensor channel. In this experiment, most of the galinstan expelled from the sensor part moved to the inlet and outlet holes punched by a 0.3-mm biopsy puncher for wire connection. Since the volumes of the inlet and outlet holes are a great deal larger than the sensor part, the resistance change due to the expelled galinstan was negligible.

### 3.3. Viscosity Measurement

To verify the feasibility of the galinstan-based pressure sensor from a practical point of view, we performed viscosity measurements of various sample solutions. DI water (viscosity of 1 cP) was used as a reference fluid. The relative viscosity of a sample fluid was estimated at room temperature (25 °C) by comparing with the calibration results of DI water. Before conducting experiment, the viscosity of DI water was confirmed using a commercial cone and plate viscometer. To estimate the viscosity of the sample fluid from the measurement results, we utilized the Poiseuille’s law (△*P* = *Q × R_h_*), where △*P* is the pressure drop, *Q* is the flow rate, and *R_h_* is the hydraulic resistance [36], which can be expressed as:

(3) $$R_{h}=\frac{12\mu l}{wh_{3}}{\left\{1-\sum_{n=1,3,5,...}^{\infty }\frac{1}{n^{5}}\frac{192h}{w\pi^{5}}\tanh(n\pi \frac{w}{2h})\right\}}^{-1}$$

where *l*, *w*, *h*, and *μ* are the channel length, width, height, and fluid viscosity, respectively. Since the geometrical parameters are fixed, the pressure only depends on the fluid viscosity at a given flow rate, and is represented by the measured voltage data. In other words, the voltage change in the pressure sensor is affected only by the viscosity of the sample fluid when the flow rate is fixed. Therefore, we could estimate the viscosity of the sample fluid by comparing the voltage change between the reference fluid (DI water) and the sample fluid at a given flow rate.

**Figure 6:**
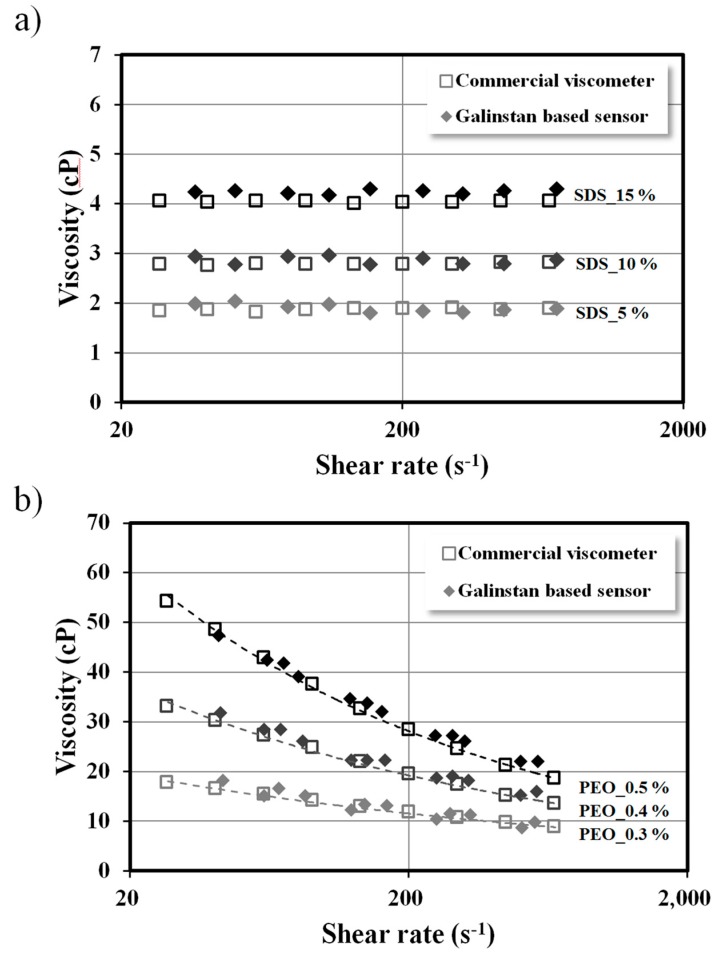
Viscosity measurement results of (**a**) 5%, 10%, and 15% SDS solution as a Newtonian fluid and (**b**) 0.3%, 0.4%, and 0.5% PEO solutions under a shear-rate range from 30 s^−1^ to 1000 s^−1^. The data measured by the galinstan-based pressure sensor are confirmed by a commercial cone and plate viscometer.

Sodium dodecyl sulfate (SDS) and polyethylene oxide (PEO) solutions were employed to measure the viscosity of the sample fluids. We prepared 5%, 10%, and 15% of SDS solution diluted with DI water as Newtonian sample fluids. Each sample fluid was injected into the fluidic channel at flow rates of 50, 150, and 450 μl/h. The viscosity of the sample fluid was simultaneously measured under three different shear-rate conditions because the device included three different channel lengths. The shear rate ($\dot{\gamma }$) is expressed as $\dot{\gamma }$ = 6*Q*/*hw*^2^, where *Q* is the flow rate, and *h* and *w* are the height and width of the fluidic channel, respectively. The flow rate in each channel can be calculated from the inlet flow rate, which was controlled by a programmable syringe pump, using the hydraulic resistance ratio of each channel. The viscosity measurement result of the Newtonian fluid is shown in Figure 6a. As we expected, the results show constant viscosity regardless of the shear rate. For the non-Newtonian fluid samples, various PEO solutions were also employed to measure the viscosity, and each sample was introduced with flow rates of 20, 100, 200, and 400 μl/h. Figure 6b shows the measurement results of 0.3%, 0.4%, and 0.5% of PEO solution samples. In contrast to the Newtonian fluid, the viscosity of the non-Newtonian fluid decreased with an increase in the shear rate. To demonstrate the results obtained using the proposed device, the viscosity of the sample fluids was evaluated by the commercial cone and plate viscometer. The normalized differences of the 5%, 10%, and 15% SDS solution were 3.76%, 3.05%, 5.02%, respectively, and those of the 0.3%, 0.4%, and 0.5% PEO solution were 6.94%, 5.91%, and 5.17%, respectively. Thus, the results measured by the proposed pressure sensor and the commercial viscometer were in good agreement. The detection limit of a pressure sensor for microviscometer application is 30 s^−1^. The proposed pressure sensor is expected to measure the viscosity of a sample fluid even at lower shear rate by further optimization. For instance, enhancement of galinstan movement, which is one of the factors that affect the signal change, can be achieved by channel surface modification using HCl [37,38] or adding a bumper structure that provides extra space for the expelled galinstan from the sensor part.

## 4. Conclusions

In this study, we investigated the performance of a liquid metal-based pressure sensor. The proposed pressure sensor was calibrated using DI water for a pressure range from 0 to 12 kPa. The results showed strong linearity (*R*^2^ > 0.999) according to the linear regression analysis. The linearity decreased with increase in the applied pressure; however, the correlation coefficient was above 0.98 for pressure up to 230 kPa. The same set of calibration was conducted for 30 days. The discrepancy between the initial calibration results and the results measured for each day was less than 5%. For further understanding, the proposed device was applied as a viscometer using various Newtonian and non-Newtonian fluids. The measurement results obtained by the galinstan-based pressure sensor were evaluated using a commercial viscometer. The normalized differences between proposed device and conventional viscometer were less than 5.1% for the Newtonian and 7.0% for the non-Newtonian fluids. The liquid metal-based pressure sensor can be easily fabricated and integrated to microfluidic systems using a simple soft lithography process. The long-term stability test demonstrated the reliability and repeatability of the proposed sensor. Therefore, the proposed pressure sensor can be employed in various microfluidic applications, as well as in microviscometers for stable pressure monitoring.
